# *IGF2BP3* Associates with Proliferative Phenotype and Prognostic Features in B-Cell Acute Lymphoblastic Leukemia

**DOI:** 10.3390/cancers13071505

**Published:** 2021-03-25

**Authors:** Artturi Mäkinen, Atte Nikkilä, Teppo Haapaniemi, Laura Oksa, Juha Mehtonen, Matti Vänskä, Merja Heinäniemi, Timo Paavonen, Olli Lohi

**Affiliations:** 1 Tampere Center for Child, Adolescent and Maternal Health Research, Faculty of Medicine and Health Technology, Tampere University, 33520 Tampere, Finland; atte.nikkila@tuni.fi (A.N.); laura.oksa@tuni.fi (L.O.); olli.lohi@tuni.fi (O.L.); 2 Fimlab Laboratories, Department of Pathology, Tampere University Hospital, 33520 Tampere, Finland; teppo.haapaniemi@fimlab.fi (T.H.); timo.paavonen@tuni.fi (T.P.); 3 Department of Biological and Environmental Sciences, University of Jyväskylä, 40014 Jyväskylä, Finland; 4 Institute of Biomedicine, School of Medicine, University of Eastern Finland, 70211 Kuopio, Finland; juha.mehtonen@uef.fi (J.M.); merja.heinaniemi@uef.fi (M.H.); 5 Department of Internal Medicine, Tampere University Hospital, 33520 Tampere, Finland; matti.vanska@pshp.fi; 6 Department of Pathology, Faculty of Medicine and Health Technology, Tampere University, 33520 Tampere, Finland; 7 Tays Cancer Centre, Tampere University Hospital, 33520 Tampere, Finland

**Keywords:** insulin-like growth factor 2 mRNA-binding protein 3 (*IGF2BP3*), mRNA, pediatric B-cell acute lymphoblastic leukemia, prognosis, proliferation, protein

## Abstract

**Simple Summary:**

Although the prognosis of acute lymphoblastic leukemia (ALL) has improved significantly during the past decades, ALL remains a major cause of pediatric cancer mortality, and more accurate risk-stratification is required. We investigated *IGF2BP3*, which has previously been associated with aggressive cancers, and found high and subtype-specific expression of *IGF2BP3* in B-cell ALL, that was associated with good outcome in high-risk patients. Results suggest that *IGF2BP3* could be useful to improve stratification and prognosis of B-ALL.

**Abstract:**

The oncofetal protein insulin-like growth factor 2 mRNA-binding protein 3 (*IGF2BP3*) belongs to a family of RNA-binding proteins involved in localization, stability, and translational regulation of target RNAs. *IGF2BP3* is used as a diagnostic and prognostic marker in several malignancies. Although the prognosis of pediatric B-cell acute lymphoblastic leukemia (B-ALL) has improved, a subgroup of patients exhibits high-risk features and suffer from disease recurrence. We sought to identify additional biomarkers to improve diagnostics, and we assessed expression of *IGF2BP3* in a population-based pediatric cohort of B-ALL using a tissue microarray platform. The majority of pediatric B-ALL cases were positive for *IGF2BP3* immunohistochemistry and were associated with an increased proliferative phenotype and activated STAT5 signaling pathway. Two large gene expression data sets were probed for the expression of *IGF2BP3*—the highest levels were seen among the B-cell lymphomas of a germinal center origin and well-established (KMT2A-rearranged and ETV6-RUNX1) and novel subtypes of B-ALL (e.g., NUTM1 and ETV6-RUNX1-like). A high mRNA for *IGF2BP3* was associated with a proliferative “metagene” signature and a high expression of *CDK6* in B-ALL. A low expression portended inferior survival in a high-risk cohort of pediatric B-ALL. Overall, our results show that *IGF2BP3* shows subtype-specificity in expression and provides prognostic utility in high-risk B-ALL.

## 1. Introduction

Pediatric B-cell acute lymphoblastic leukemia (B-ALL) is the most common malignancy in childhood. Despite the significantly improved prognosis, a subgroup of patients with either a poor therapy response or high-risk features still often experience a relapse. Better diagnostic tools are needed to enhance treatment stratification and prognosis, and to avoid overtreatment and adverse long-term side-effects [1,2,3].

Insulin-like growth factor II mRNA-binding protein 3 (*IGF2BP3*), also known as the *IGF2BP3* protein, is a 69 kDa protein that localizes mostly to the cytoplasm [4,5]. This oncofetal RNA-binding protein is a member of the IGF2BP-family, which also includes IGF2BP1 and IGF2BP2 proteins, and shares 59–73% similarity with the amino acid sequence with *IGF2BP3* [6,7]. *IGF2BP3* binds RNA molecules and acts as a regulator of mRNA localization and stability [7,8]. It is expressed only at a low level in most adult tissues, whereas in multiple human malignancies, it is overexpressed [7,8].

Mutations of *IGF2BP3* are rare, but the expression is dysregulated at epigenetic, transcriptional, and post-transcriptional levels. At a cellular level, *IGF2BP3* drives miRNA biogenesis; intercepts the cytoplasmic export of mRNA; and regulates mRNA stability, degradation, and transportation [7]. In gastrointestinal and urogenital malignancies, *IGF2BP3* is highly expressed, and is associated with cell adhesion, tumor invasion, metastasis, and inferior outcomes [7,8,9]. *IGF2BP3* exhibits a strong expression in lymphoid malignancies such as B cell lymphomas of a germinal center origin [10,11]. It is expressed in Reed–Sternberg cells and can be used as a supplementary diagnostic marker in Hodgkin’s lymphoma [12,13,14]. Increased expression of *IGF2BP3* is associated with proliferative features in many solid tumors, mantle cell lymphoma, and chronic myeloid leukemia blast crisis [7,15,16], and promotes cell survival during ionizing radiation in B-cells [17].

Stoskus et al. [18] explored the expression of IGF2BP family members in hematopoietic tissues and ALL by using isoform-specific RT-qPCR. In healthy stem or mature hematopoietic cells, the expression of *IGF2BP3* was either weak or absent in contrast to *IGF2BP2*. The analysis of different mature cell populations demonstrated that only CD19+ B-cells expressed detectable levels of *IGF2BP3*, in line with previous literature [10,18,19]. Among B-ALL, the strongest expression was evident in ETV6-RUNX1 and KMT2A-rearranged subtypes. Liao et al. [16] and Palanichamy et al. [20] showed that siRNA or CRISPR-Cas9-mediated the knockdown of *IGF2BP3* reduced proliferation and increased apoptosis in several cell lines (K562, RS4;11, and NALM6).

While a growing body of data supports biological significance and prognostic utility of *IGF2BP3* in different epithelial and soft tissue tumors, to date, there are only two studies that have explored its expression in lymphoid leukemias [18,20], and no studies that have assessed expression at the protein level. Hence, we investigated the expression of *IGF2BP3* across hematological malignancies and in a trephine biopsy sample cohort of pediatric B-ALL and correlated its expression with cell proliferative features and patient survival.

## 2. Materials and Methods

### 2.1. Patient Cohort for Tissue Microarray and Immunohistochemistry

The formalin-fixed and paraffin-embedded bone marrow trephine biopsy samples of the pediatric B-ALL patients were collected into a tissue microarray (TMA) with 1.5 mm punches (see also [21]), and 4-micrometer TMA sections were used for immunohistochemistry. An appendix was used as a control material for the *IGF2BP3* and CD19/Ki-67 immunostainings. Immunohistochemistry was performed using the Ventana Benchmark Ultra instrument. BCL6 and pSTAT5 (Y694) immunohistochemistry was performed on whole tissue sections using Ventana Benchmark Classic [22]. The following antibodies were used: *IGF2BP3* (lot: 11085707, clone: 69.1, manufacturer: Dako, Santa Clara, CA, USA, id: M3626, dilution: 1:100, species: mouse monoclonal, Ig class: IgG2a, kappa), CD19 (lot: 000085227, clone: EP169, manufacturer: Cell Marque, Rocklin, CA, USA, id: 119R-18, dilution: ready-to-use, species: rabbit monoclonal, Ig class: IgG), Ki-67 (lot: F30644, clone: 30-9, manufacturer: Ventana, Tucson, AZ, USA, id: 790-4286, dilution: ready-to-use, species: rabbit monoclonal, Ig class: IgG), BCL6 (lot: 48794, clone: LN22, id: PA0204, species: mouse monoclonal, manufacturer: Leica Biosystems, Newcastle, UK, dilution: 1:50), and pSTAT5 (Y694) (lot: GR208043, clone: E208, id: ab32364, manufacturer: Abcam, Cambridge, UK, dilution: 1:50). For the *IGF2BP3* and Ki-67 stainings, we used the OptiView DAB detection kit; for the CD19 stainings, the UltraView Universal Alkaline Phosphatase Red detection kit; and for BCL6 and pSTAT5 (Y694), the Ultraview Universal DAB detection kit. All of the slides were counterstained using hematoxylin. The expression of BCL6 and pSTAT5 was semiquantitatively graded as negative when antigen was expressed in under 20% of leukemic blasts, and positive when expressed in over 20%. Clinical data and the flow cytometry data (e.g., CD34 expression) were retrieved from patient hospital records gathered as described previously [21]. The flow cytometry results were graded as either negative or positive.

### 2.2. Image Analysis

Slides were scanned with Hamamatsu Nanozoomer XR using 40× magnification. QuPath software (version 0.2.3) [23] was used to detect cytoplasmic *IGF2BP3* positivity in TMA-sections from annotated areas with leukemic cells. A pathologist manually set detection parameters and thresholds using the cytoplasmic staining of *IGF2BP3* in germinal center cells as a reference, and the nuclear staining in germinal centers and proliferating epithelium as a reference for Ki-67 staining. The stain vectors and intensity thresholds for the cell and antibody detection were adjusted according to the instructions of the QuPath software in visual control. Inadequate samples were removed from the analysis. Areas with artifacts caused by compression or folding of the tissue were disregarded by setting the proper threshold values for background intensity. With the *IGF2BP3* and CD19/Ki-67- double-stained slides, stain vectors were adjusted for hematoxylin, 3,3′-diaminobenzidine (DAB), and alkaline phosphatase (AP) staining using a representative region of interest. Hematoxylin-stained cells were detected using the cell detection function in the QuPath, while the nuclear DAB of Ki-67-positive cells were recognized from the CD19-positive (AP) areas. Single intensity thresholds for *IGF2BP3*, CD19, and Ki-67 were used to assess the proportion of positive cells.

### 2.3. Microarray and RNA-Sequencing Data Sets

Hemap is a microarray gene expression data set that includes 6832 cancer samples and 1304 B-ALL samples (662 pediatric and 642 adult cases) [24,25]. The RNA-sequencing data set from the PanALL study cohort includes 1988 B-ALL cases (1234 pediatric and 754 adult cases) [26]. For the survival analyses, the TARGET data set, which includes 155 cases of pediatric high-risk B-ALL patients, was retrieved along with the following clinical information: events (relapse, induction failure, death, and second malignancy), survival, age, leukocyte count, minimal residual disease (MRD) at the end of induction (EOI), and the cytogenetic subtype [27,28].

### 2.4. Statistical Analysis

The statistical analysis was conducted using IBM SPSS Statistics (version 26) and RStudio (version 3.6.1). The Mann–Whitney U test, Kruskal–Wallis U test, chi-squared test, Fisher’s exact, and log-rank test were used to test the significance of the differences between groups. All tests were two-sided, and *p*-values under 0.05 were considered statistically significant. The ComplexHeatmap package in R was used to create heatmaps [29]. Cox proportional hazards models were fitted for survival data in order to estimate the hazard of individual risk factors.

## 3. Results

### 3.1. IGF2BP3 Protein Is Widely Expressed in Pediatric B-ALL

**Figure 1:**
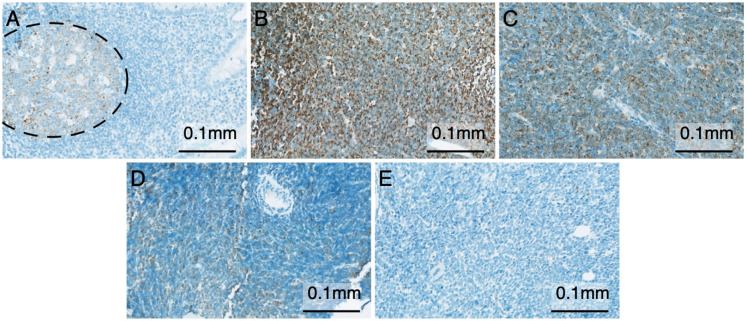
Immunohistochemistry of insulin-like growth factor II mRNA-binding protein 3 (*IGF2BP3*). (**A**) Appendix showing positivity (brown color) to *IGF2BP3* in the germinal center (dashed circle; 200× magnification). (**B**) Strongly *IGF2BP3*-positive bone marrow trephine biopsy of a B-cell acute lymphoblastic leukemia (B-ALL) patient (200× magnification). (**C**) Pediatric B-ALL case with a heterogeneous pattern of *IGF2BP3* expression (200× magnification). (**D**) Weakly *IGF2BP3*-positive B-ALL case with only singular positive cells visible (200× magnification). (**E**) *IGF2BP3*-negative B-ALL case (200× magnification).

The *IGF2BP3* protein has shown diagnostic and prognostic utility in different malignancies [7]. To assess the expression of the *IGF2BP3* protein in B-ALL, we employed a population-based pediatric cohort of 83 B-ALL cases, and immunostained the diagnostic bone marrow trephine biopsies embedded in a tissue microarray (TMA) with an antibody against *IGF2BP3*. The case summary for the TMA samples is shown in Table 1. The appendix was used as a positive control, and it was stained positively in the germinal centers of the lymphoid follicles, as expected (Figure 1A) [10]. Positivity (>1%) to *IGF2BP3* was detected in 74 out of 83 patients (89%; Figure 1B–D), while the proportion of positively stained leukemia cells ranged from 1 to 100% (median 34%). *IGF2BP3* exhibited a granular staining pattern and was localized mostly to the cytoplasm. Negative *IGF2BP3* staining was found in 9 out of 83 B-ALL cases (Figure 1E). No expression of *IGF2BP3* was found in the remission bone marrow specimens.

**Figure 2:**
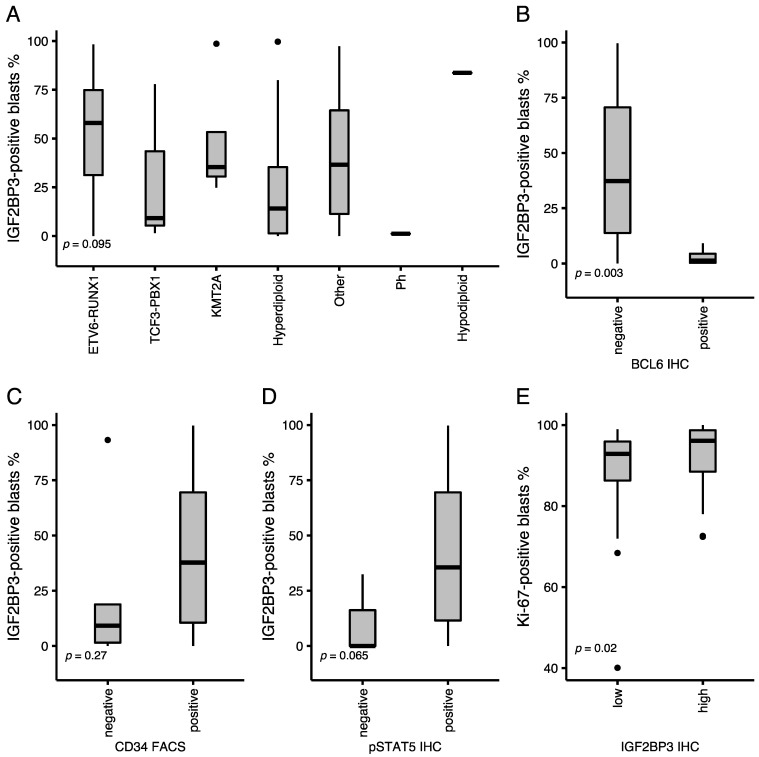
Immunophenotype of a B-cell acute lymphoblastic leukemia (B-ALL) tissue microarray cohort. (**A**) Expression of *IGF2BP3* according to the WHO classification of B-ALL. (**B**) Positivity to *IGF2BP3* among cases with either a negative or positive expression of the BCL6 protein. (**C**) Positivity to *IGF2BP3* in cases with negative or positive CD34. (**D**) Positivity to *IGF2BP3* in cases with a negative or positive pSTAT5 (Y694). (**E**) Expression of Ki-67 among cases with either a low or high *IGF2BP3* (median as a cut-off). Dots depict outliers. *p*-values of (**B**–**E**) Mann–Whitney U test and (**A**) Kruskal–Wallis test are shown.

We classified the cases into distinct subtypes according to the WHO 2017 Classification of B-ALL [30]. Expression of *IGF2BP3* protein was highest in the ETV6-RUNX1, “Other”, KMT2A-rearranged, and hypodiploid subtypes (Figure 2A). The difference was statistically significant between ETV6-RUNX1 and other subtypes (Mann Whitney U Test *p*-value = 0.04). The expression of *IGF2BP3* protein did not correlate with white blood cell count (WBC), MRD at the end of induction (EOI), CNS disease, or expression of specific cell surface markers.

*IGF2BP3* is normally expressed in germinal centers, where the BCL6 protein is active and associated with germinal center-type B-cell lymphomas [10]. We recently showed that the BCL6 protein is also expressed in a fraction of precursor B-ALL [22]. BCL6-positivity, CD34-negativity, and pSTAT5-negativity have been associated with a novel pre-B-cell receptor signaling subtype of B-ALL [31]. Hence, we tested the association between *IGF2BP3* and BCL6 proteins and discovered that the *IGF2BP3* protein was significantly lower among the BCL6-positive cases (Mann–Whitney U test; *p*-value = 0.003). Likewise, the mRNA expression of the pre-BCR “metagene” (see below), which is associated with BCL6-positivity [31], exhibited a significantly lower expression among the highest 10th percentile of the *IGF2BP3* expressing patients in the PanALL and Hemap data sets (Mann–Whitney U; *p*-value < 0.001). On the contrary, cases that exhibited phosphorylated STAT5 (pY694) protein or showed a high expression of the stem cell marker CD34 evidenced a higher-than-median level of the *IGF2BP3* protein (Figure 2B–D).

### 3.2. Expression of Ki-67 Is Associated with High IGF2BP3 Protein Expression

A high expression of *IGF2BP3* has been associated with proliferative phenotype in malignancies such as mantle cell lymphoma [7,15]. We assessed whether it is associated with cell proliferation in B-ALL by co-staining the trephine biopsy specimens with CD19, a marker of blast cells, and Ki-67, a well-established marker of cell proliferation [32,33]. Overall, the expression of Ki-67 was strong in proliferating cells of germinal centers and the epithelium of appendix (Figure S1A), and in CD19-positive cells of B-ALL samples (Figure S1B,C; proportion of positive cells, median 95%, interquartile range (IQR) 87–98%). A higher-than-median level of *IGF2BP3* was significantly associated with the expression of Ki-67 (Mann–Whitney U test; *p*-value = 0.02; Figure 2E).

### 3.3. Expression of IGF2BP3 in Hematological Malignancies and B-ALL

**Figure 3:**
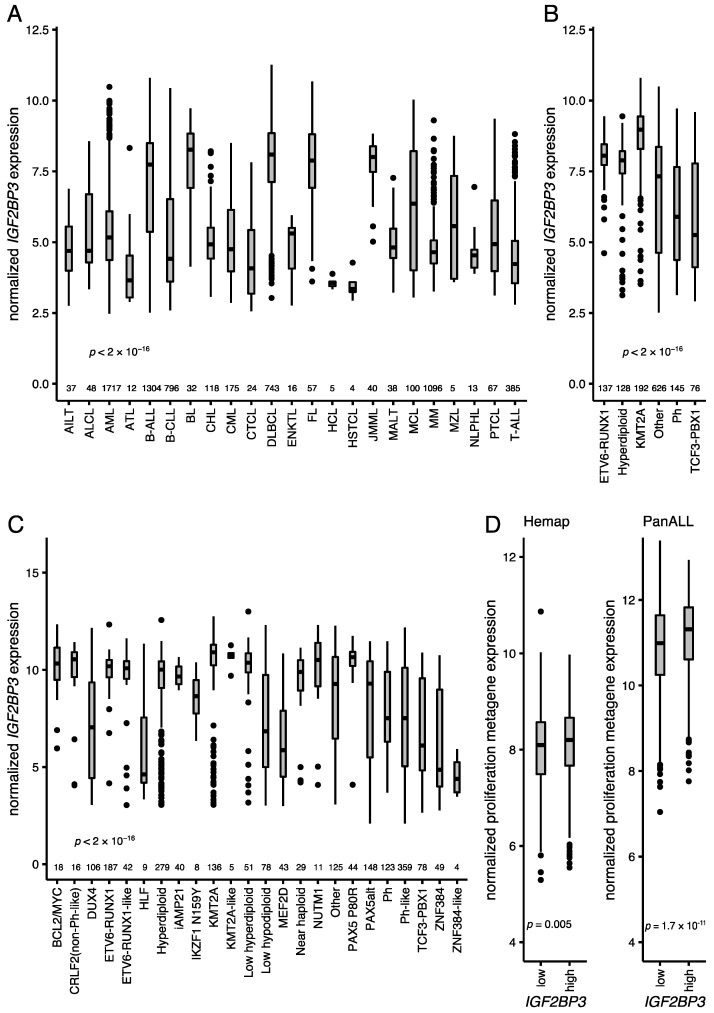
Expression of *IGF2BP3* across different hematological malignancies and subtypes of B-ALL. (**A**) *IGF2BP3* expression in the Hemap data set in different hematological malignancies (*n* = 6832) [24,25]. (**B**) *IGF2BP3* expression in different cytogenetic subtypes of B-ALL (*n* = 1304) in the Hemap data set [24,25]. (**C**) *IGF2BP3* expression in different B-ALL subtypes of B-ALL in the PanALL study cohort (*n* = 1988) [26]. (**D**) Proliferation-associated “metagene” [33] expression in B-ALL in the Hemap and PanALL data sets (median as a cut-off for the *IGF2BP3* expression groups). AILT—angioimmunoblastic T-cell lymphoma; ALCL—anaplastic large cell lymphoma; AML—acute myeloid leukemia; ATL—adult T-cell leukemia; B-ALL—B-cell lineage acute lymphoblastic leukemia; B-CLL—B-cell chronic lymphocytic leukemia; BCL2/MYC—*BCL2/MYC*-rearranged; BL—Burkitt lymphoma; CHL—classic Hodgkin lymphoma; CML—chronic myeloid leukemia; CRLF2—*CRLF2* (non-Ph-like); CTCL—cutaneous T-cell lymphoma; DLBCL—diffuse large B-cell lymphoma; DUX4—*DUX4*-rearranged; ENKTL—extranodal NK/T-cell lymphoma; FL—follicular lymphoma; HCL—hairy cell leukemia; HLF—*TCF3/TCF4-HLF*; HSTCL—hepatosplenic T-cell lymphoma; iAMP21—intrachromosomal amplification of chromosome 21; IKZF1 N159Y—*IKZF1* missense alteration encoding p.Asn159Tyr; JMML—juvenile myelomonocytic leukemia; KMT2A—KMT2A-rearranged; MALT—extranodal marginal zone lymphoma of mucosa-associated lymphoid tissue; MCL—mantle cell lymphoma; MEF2D—*MEF2D*-rearranged; MM—multiple myeloma; MZL—marginal zone lymphoma; *n*—number of cases; NLPHL—nodular lymphocyte predominant Hodgkin lymphoma; NUTM1—*NUTM1*-rearranged; PAX5alt—PAX5 alterations; PAX5 P80R—PAX5 p.Pro80Arg (P80R) alteration; Ph—Philadelphia chromosome (*BCR-ABL1*); PTCL—peripheral T-cell lymphoma, not otherwise specified; T-ALL—T-cell lineage acute lymphoblastic leukemia; ZNF384—*ZNF384*-rearranged. Dots depict outliers. *p*-values of (**D**) Mann–Whitney U test and (**A**–**C**) Kruskal–Wallis test are shown.

*IGF2BP3* is associated with various malignancies of a B-cell origin, and particularly with germinal center lymphomas [10,11]. To get a comprehensive picture across hematological tumors, we assessed *IGF2BP3* mRNA levels in 6832 hematological cancers that included 24 different disease entities [24,25]. The median expression of the *IGF2BP3* mRNA was the highest in B-ALL, Burkitt lymphoma, diffuse large B-cell lymphoma, follicular lymphoma, mantle cell lymphoma, and juvenile myelomonocytic leukemia, while the lowest median expressions were observed in hairy cell leukemia, hepatosplenic T-cell lymphoma, and adult T-cell leukemia (Figure 3A). The *IGFBP3* mRNA was present in all subtypes of B-ALL, with the highest expression in the KMT2A-rearranged and ETV6-RUNX1 subtypes and the lowest in the TCF3-PBX1 and BCR-ABL1 subtypes (Figure 3B). Analysis of the PanALL data set [26], which comprises 1988 B-ALL cases, validated the findings, and also revealed a strong expression in novel subtypes such as NUTM1-rearranged, PAX5-altered, ETV6-RUNX1-like, BCL2/MYC, and CRLF2 (Figure 3C).

### 3.4. Proliferative “Metagene” Signature in B-ALL

Recently, Giuliano et al. (2018) [33] described a proliferative “metagene” (*MKI67*, *PCNA*, *CCNB1*, *MCM2*, and *TOP2A*) that is correlated with cell proliferation. Supporting our earlier observations, the *IGF2BP3* mRNA was significantly associated with the proliferative “metagene” when assessed across all hematological malignancies (Mann–Whitney U test; *p*-value < 0.001). When the analysis was restricted to the B-ALL cases, the “metagene” signature and the *MKI67* mRNA showed elevated levels and were significantly associated with a higher-than-median expression of *IGF2BP3* (Mann–Whitney U test; *p*-value = 0.005 and *p*-value = 0.04, respectively; Figure 3D). A similar analysis in the PanALL data set replicated the findings: the *IGF2BP3* mRNA was significantly associated with the high *MKI67* mRNA (Mann–Whitney U test, *p*-value < 0.001) and the proliferation-associated “metagene” with a discretized expression of *IGF2BP3* (Mann–Whitney U test, *p*-value < 0.001; Figure 3D; see also heatmap in Figure S2A–C).

*CDK6* and MYC oncoproteins have been reported as targets of the *IGF2BP3* protein [20]. In the PanALL data set, *CDK6* was higher and *MYC* was lower among cases with a higher-than-median *IGF2BP3* mRNA (Mann–Whitney U test *p*-value < 0.001, Figure S3A,B). In the Hemap data set, *CDK6* was strongly expressed among cases with a higher-than-median *IGF2BP3* mRNA, whereas the expression of *MYC* did not differ (Figure S3C,D).

### 3.5. High IGF2BP3 mRNA Associates with Favorable Survival in High-Risk B-ALL

**Figure 4:**
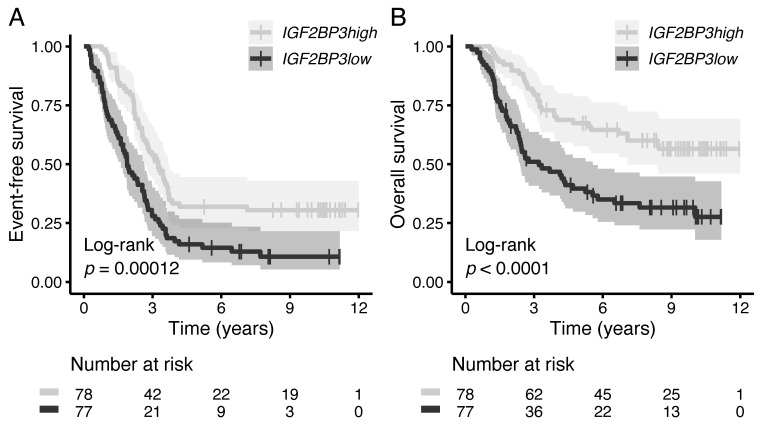
Association of *IGF2BP3* expression at an mRNA level on patient survival. Kaplan–Meier survival analysis for (**A**) event-free survival and (**B**) overall survival in the high-risk B-ALL TARGET cohort (*n* = 155) [27,28]. Statistical significance was tested using the log-rank test, while the median expression of *IGF2BP3* was used as a cut-off for the two different patient groups.

The prognostic value of *IGF2BP3* mRNA was evaluated in the TARGET data set that included high-risk pediatric B-ALL cases [27,28]. Higher-than-median *IGF2BP3* mRNA showed a statistically significant association with favorable event-free (EFS) and overall survival (OS; Figure 4A,B). In a multivariate analysis that included age, white blood cell count (WBC), and minimal residual disease (MRD) at the EOI as covariates, higher-than-median *IGF2BP3* mRNA exhibited a decreased hazard ratio for events (HR 0.46, 95% CI 0.31–0.68) and death (HR 0.50, 95% CI 0.31–0.81; Table 2).

In the population-based TMA cohort, positivity to *IGF2BP3* protein did not associate with patient survival (data not shown).

## 4. Discussion

*IGF2BP3* is an oncofetal protein that is normally expressed in fetal/embryonic tissues, but is often aberrantly re-expressed in malignant tumors. In solid tumors, its expression is associated with increased proliferation and inferior outcomes. We report here that *IGF2BP3* is widely expressed in pediatric B-ALL, and shows a granular staining pattern fitting to the cytoplasmic ribonucleoprotein (RNP) complexes. Its expression is associated with proliferative features and a high level of *CDK6*, while a low expression confers inferior outcomes in a cohort of high-risk B-ALL patients.

Earlier gene expression studies have associated *IGF2BP3* with the ETV6-RUNX1 and KMT2A-rearranged subtypes of B-ALL [18,20]. This aligns well with our results, which were based on large gene expression microarray (Hemap) and RNA-sequencing data sets (PanALL). As novel findings, we report here a strong expression of *IGF2BP3* in new subtypes of B-ALL, such as ETV6-RUNX1-like, KMT2A-like, and NUTM1. Moreover, we extended analyses across the whole spectrum of hematological malignancies covering 24 diseases: B cell lymphomas were the strongest expressors, followed by juvenile myelomonocytic leukemia and mantle cell lymphoma, while most diseases of T-cell origin showed a lower expression.

At the protein level, our data are unique. We developed a population-based TMA platform that included 83 pediatric B-ALL cases. Almost 90% of the cases showed moderate or strong positivity to *IGF2BP3*, often in conjunction with a marker of activated JAK-STAT signaling (pSTAT5A-Y694). Fittingly, the expression of the BCL6 protein, which is not exhibited simultaneously with the activated JAK-STAT5 pathway [34], was absent among *IGF2BP3*-positive cases. The intracellular staining pattern of *IGF2BP3* was granular, possibly referring to the localization of *IGF2BP3* to cytoplasmic RNP complexes, where it exerts its function on the target mRNAs. One limitation of the immunohistochemistry results is the possibility of the cross-reactivity of the used *IGF2BP3* antibody (and other commercial *IGF2BP3* antibodies) with the paralogs of IGF2BP family [7,35,36].

By using the TMA platform, we performed co-stainings with a proliferative marker for Ki-67 and CD19, and showed that the *IGF2BP3* protein is associated with active cellular proliferation. This is not a surprise, as B-ALL is an aggressive malignancy with a high proliferative capacity. At the mRNA level, the proliferation-associated “metagene” signature was higher in patients with a strong expression of *IGF2BP3*. Although the absolute differences were small, the results fit well with earlier data in mantle cell lymphoma and solid tumors, where the expression of *IGF2BP3* was similarly associated with proliferation [7,15]. Likewise, a study by Palanichamy et al. [20] showed that the exogenous expression of *IGF2BP3* increased the proliferation of bone marrow progenitor cells and provided them with a competitive survival advantage, and that *IGF2BP3* was essential for the survival of several B-ALL cell lines. Overall, *IGF2BP3* seems to play an active role in the proliferative capacity of B cell blasts, a feature that could possibly be utilized for diagnostic or therapeutic purposes.

We correlated the expression of *IGF2BP3* with the patient outcome in a high-risk pediatric B-ALL cohort, in which a higher-than-median *IGF2BP3* mRNA level was associated with improved survival. However, no association with outcome was evident for the *IGF2BP3* protein in our population-based biopsy cohort, possibly because of its relatively small size or low number of events. A recent single cell analysis of early therapy response in B-ALL showed that patients with high proliferative features are more sensitive to induction chemotherapy, and that therapy-resistant clones are more likely among the quiescent cell populations [37]. Hence, *IGF2BP3* expression is associated with cell populations that are actively dividing and more are likely to be killed by chemotherapy, which is reflected in the overall outcome of patients. We note that the prognostic effect was evident only in the high-risk cohort, and therefore in the future, outcome data need further exploration in larger population-based data sets.

## 5. Conclusions

In conclusion, we found that a high expression of *IGF2BP3* is associated with a proliferative phenotype in pediatric B-ALL at mRNA and protein levels, and portends a favorable survival high-risk B-ALL. Our results show that the subtype-specific expression of *IGF2BP3* provides diagnostic and prognostic utility in B-ALL.

## Figures and Tables

**Table 1:** Case summary of the tissue microarray (TMA) cohort.

Clinical Parameter	Median (IQR)
Age (years)	4.3 (2.7–9.7)
WBC (x 10E9/l)	6.3 (2.7–29.2)
MRD (%), EOI	0.01 (0.00–0.14)
	***n* (%)**
CNS disease	5 (6.0)
Total	83
**WHO Subtype**	
Other	32 (38.6)
BCR-ABL1	1 (1.2)
KMT2A-re	4 (4.8)
ETV6-RUNX1	20 (24.1)
Hyperdiploid	22 (26.5)
Hypodiploid	1 (1.2)
TCF3-PBX1	3 (3.6)

EOI—end of induction; IQR—interquartile range; KMT2A-re—KMT2A-rearranged; MRD—minimal residual disease; WBC—white blood cell count; WHO—World Health Organization.

**Table 2:** Cox proportional hazards model for survival in the high-risk B-ALL TARGET cohort (*n* = 155) [27,28].

Event-Free Survival							
		Univariate			Multivariate		
		HR	95% CI	*p*	HR	95% CI	*p*
*IGF2BP3* mRNA	≤median	1			1		
	>median	0.49	0.34–0.71	<0.001	0.46	0.31–0.68	<0.001
Age		1.01	0.97–1.05	0.69	0.97	0.93–1.01	0.19
MRD at the EOI		1.02	0.98–1.07	0.39	1.01	0.97–1.06	0.58
Overall survival							
		Univariate			Multivariate		
		HR	95% CI	*p*	HR	95% CI	*p*
*IGF2BP3* mRNA	≤median	1			1		
	>median	0.44	0.28–0.68	<0.001	0.5	0.31–0.81	0.006
Age		1.06	1.01–1.11	0.01	1.03	0.98–1.08	0.24
WBC		1	1.00–1.00	0.58	1	0.99–1.00	0.68
MRD at the EOI		1.02	0.97–1.08	0.47	0.99	0.94–1.05	0.79

CI—confidence interval; EOI—end of induction therapy; HR—hazards ratio; MRD—minimal residual disease; WBC—white blood cell count at diagnosis.

## Data Availability

The results published here are in whole or part based upon data generated by the Therapeutically Applicable Research to Generate Effective Treatments (https://ocg.cancer.gov/programs/target) initiative, phs000463. The data used for this analysis are available at https://portal.gdc.cancer.gov/projects (accessed on 27 August 2020).

## Supplementary Materials

The following are available online at https://www.mdpi.com/2072-6694/13/7/1505/s1, Figure S1: Immunohistochemical co-staining of CD19 and Ki-67 in B-ALL, Figure S2: Heatmap illustration of expression of proliferation-associated genes (*MKI67*, *PCNA*, *CCNB1*, *MCM2*, and *TOP2A*) in cases with either a high or low expression of *IGF2BP3*, Figure S3: Boxplots showing expression of *CDK6* and *MYC* in cases with either a low or high expression of *IGF2BP3* in B-ALL.
