# Supplementary material for: IGF2BP3 Associates with Proliferative Phenotype and Prognostic Features in B-Cell Acute Lymphoblastic Leukemia
Source: Cancers (Basel). 2021 Mar 25;13(7):1505. doi: 10.3390/cancers13071505 (PMC8037952; doi:10.3390/cancers13071505)
Supplement: Supplementary file 1 [file cancers-13-01505-s001.pdf]

cancers

MDPI

Article

IGF2BP3 Associates with Proliferative Phenotype and Prognostic Features in B-Cell Acute Lymphoblastic Leukemia

Artturi Mäkinen [1,2,*], Atte Nikkilä [1], Teppo Haapaniemi [2,3], Laura Oksa [1], Juha Mehtonen [4], Matti Vänskä [5], Merja Heinäniemi [4], Timo Paavonen [2,6] and Olli Lohi [1,7]

1 Tampere Center for Child, Adolescent and Maternal Health Research, Faculty of Medicine and Health Technology, Tampere University, 33520 Tampere, Finland; atte.nikkila@tuni.fi (A.N.); laura.oksa@tuni.fi (L.O.); olli.lohi@tuni.fi (O.L.)
2 Fimlab Laboratories, Department of Pathology, Tampere University Hospital, 33520 Tampere, Finland; teppo.haapaniemi@fimlab.fi (T.H.); timo.paavonen@tuni.fi (T.P.)
3 Department of Biological and Environmental Sciences, University of Jyväskylä, 40014 Jyväskylä, Finland
4 Institute of Biomedicine, School of Medicine, University of Eastern Finland, 70211 Kuopio, Finland; juha.mehtonen@uef.fi (J.M.); merja.heinaniemi@uef.fi (M.H.)
5 Department of Internal Medicine, Tampere University Hospital, 33520 Tampere, Finland; matti.vanska@pshp.fi
6 Department of Pathology, Faculty of Medicine and Health Technology, Tampere University, 33520 Tampere, Finland
7 Tays Cancer Centre, Tampere University Hospital, 33520 Tampere, Finland
* Correspondence: artturi.makinen@tuni.fi

Citation: Mäkinen, A.; Nikkilä, A.; Haapaniemi, T.; Oksa, L.; Mehtonen, J.; Vänskä, M.; Heinäniemi, M.; Paavonen, T.; Lohi, O. IGF2BP3 Associates with Proliferative Phenotype and Prognostic Features in B-Cell Acute Lymphoblastic Leukemia. *Cancers* **2021**, *13*, 1505. https://doi.org/10.3390/cancers13071505.

Academic Editor: Ajay Pratap Singh

Received: 13 February 2021
Accepted: 22 March 2021
Published: 25 March 2021

Publisher's Note: MDPI stays neutral with regard to jurisdictional claims in published maps and institutional affiliations.

Copyright: © 2021 by the authors. Licensee MDPI, Basel, Switzerland. This article is an open access article distributed under the terms and conditions of the Creative Commons Attribution (CC BY) license (http://creativecommons.org/licenses/by/4.0/).

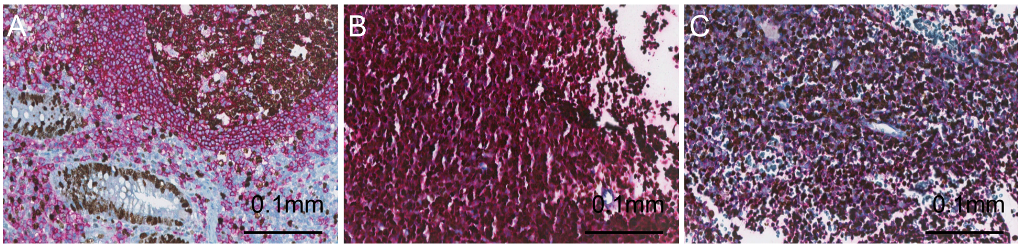

Figure S1. Immunohistochemical co-staining of CD19 and Ki-67 in B-ALL. (**A**) CD19 (red) and Ki-67 (brown) in the appendix show a high expression of Ki-67 in the germinal center (upper right) and crypt epithelium (lower left; 200X magnification). (**B**) Co-staining of CD19 and Ki-67 in the B-ALL case with a high proportion of blasts showing positivity to nuclear Ki-67 staining. (**C**) B-ALL case showing partial expression of the nuclear Ki-67 protein (200X magnification).

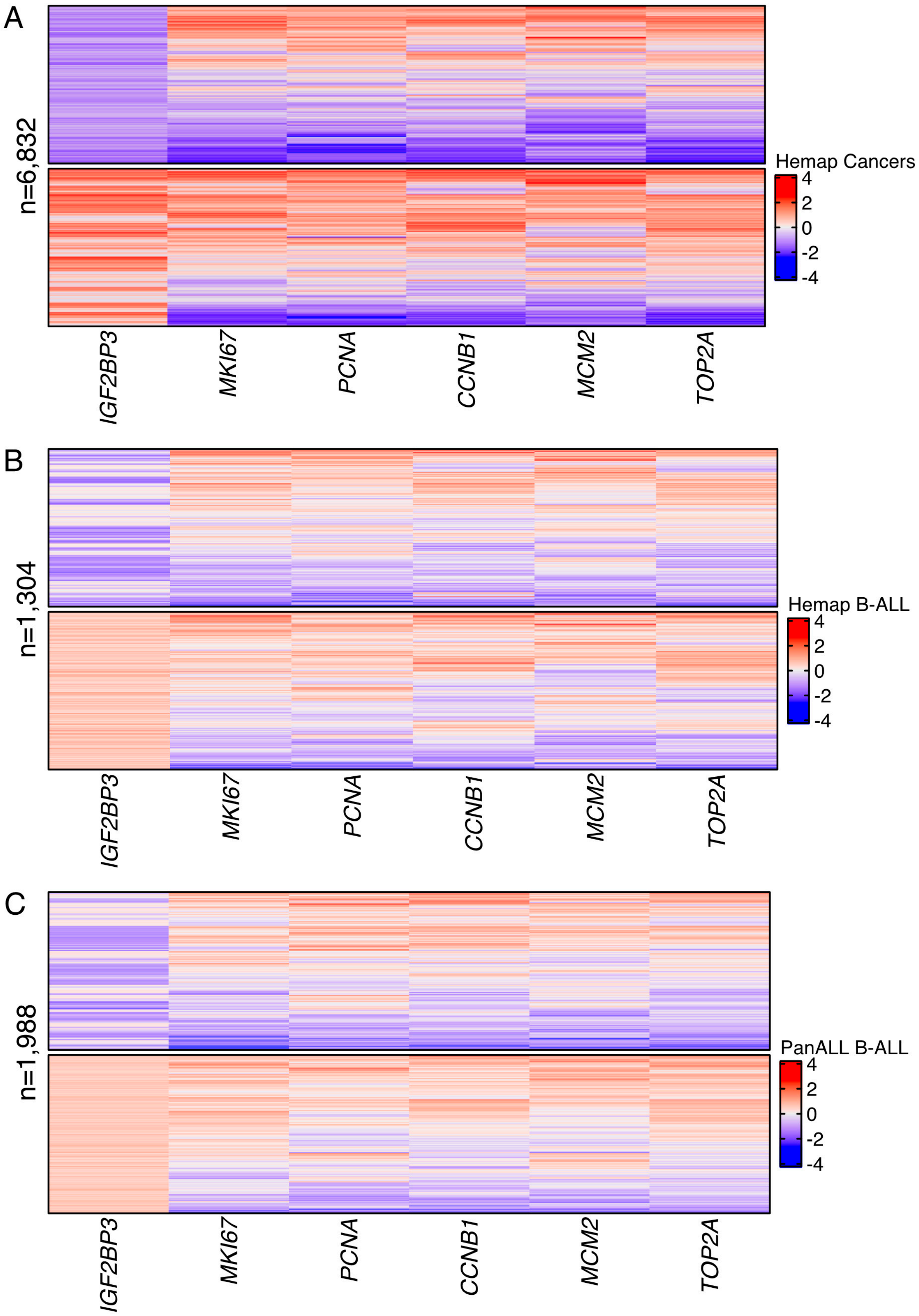

Figure S2. Heatmap illustration of the expression of proliferation-associated genes (*MKI67*, *PCNA*, *CCNB1*, *MCM2*, and *TOP2A*) in cases with either a high or low expression of *IGF2BP3* [29]. (**A**) Heatmap illustrations using (**A**) all hematological cancers or (**B**) B-ALL from the Hemap data set [24,25], and (**C**) B-ALL cases from the PanALL data set [26].

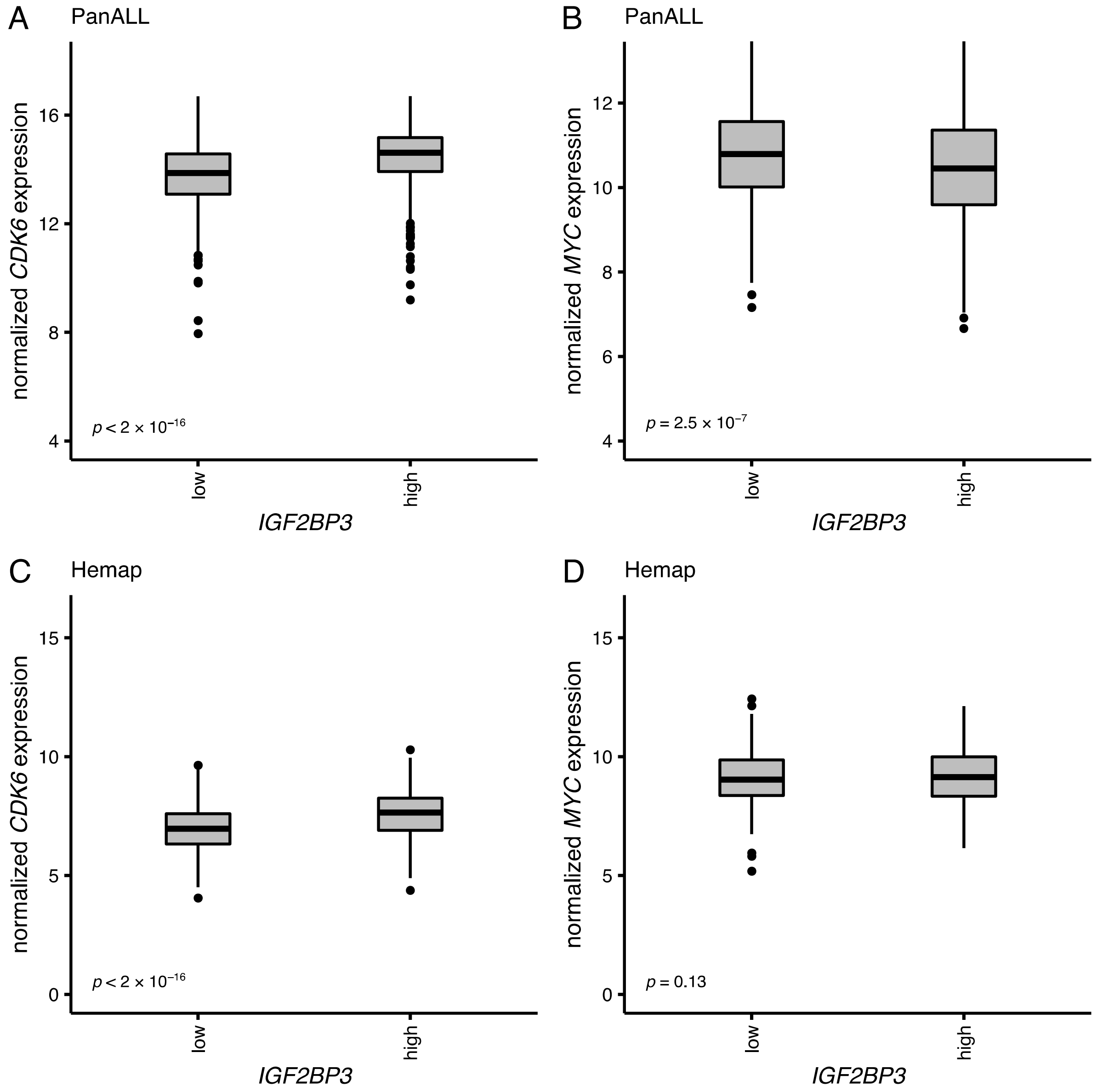

Figure S3. Boxplots showing expression of *CDK6* and *MYC* in cases with either a low or high expression of *IGF2BP3* in B-ALL for both of the data sets: (**A**,**B**) PanALL data set and (**C**,**D**) Hemap data set [24–26]. Dots represent outliers. The *p*-values of the Mann–Whitney U tests between groups are shown.
